# Contrast Adaptation in Face Perception Revealed Through EEG and Behavior

**DOI:** 10.3389/fnsys.2021.701097

**Published:** 2021-10-29

**Authors:** O. Scott Gwinn, Talia L. Retter, Sean F. O’Neil, Michael A. Webster

**Affiliations:** ^1^Visual Perception Lab, Department of Psychology, University of Nevada, Reno, NV, United States; ^2^Cognitive Neuroscience Lab, Department of Behavioural and Cognitive Sciences, Institute of Cognitive Science & Assessment, University of Luxembourg, Esch-sur-Alzette, Luxembourg

**Keywords:** EEG, periodic stimulation, face perception, adaptation, contrast, variance

## Abstract

Exposure to a face can produce biases in the perception of subsequent faces. Typically, these face aftereffects are studied by adapting to an individual face or category (e.g., faces of a given gender) and can result in renormalization of perceptions such that the adapting face appears more neutral. These shifts are analogous to chromatic adaptation, where a renormalization for the average adapting color occurs. However, in color vision, adaptation can also adjust to the variance or range of colors in the distribution. We examined whether this variance or contrast adaptation also occurs for faces, using an objective EEG measure to assess response changes following adaptation. An average female face was contracted or expanded along the horizontal or vertical axis to form four images. Observers viewed a 20 s sequence of the four images presented in a fixed order at a rate of 6 Hz, while responses to the faces were recorded with EEG. A 6 Hz signal was observed over right occipito-temporal channels, indicating symmetric responses to the four images. This test sequence was repeated after 20 s adaptation to alternations between two of the faces (e.g., horizontal contracted and expanded). This adaptation resulted in an additional signal at 3 Hz, consistent with asymmetric responses to adapted and non-adapted test faces. Adapting pairs have the same mean (undistorted) as the test sequence and thus should not bias responses driven only by the mean. Instead, the results are consistent with selective adaptation to the distortion axis. A 3 Hz signal was also observed after adapting to face pairs selected to induce a mean bias (e.g., expanded vertical and expanded horizontal), and this signal was not significantly different from that observed following adaption to a single image that did not form part of the test sequence (e.g., a single image expanded both vertically and horizontally). In a further experiment, we found that this variance adaptation can also be observed behaviorally. Our results suggest that adaptation calibrates face perception not only for the average characteristics of the faces we experience but also for the gamut of faces to which we are exposed.

## Introduction

The appearance of a face can be strongly affected by the faces seen previously, and many studies have examined the properties and implications of these face adaptation effects (Rhodes et al., 2005; Webster and MacLeod, 2011; Mueller et al., 2020). The strength of adaptation as an investigative tool stems from the recognition that the resulting perceptual aftereffects reflect changes in the response properties of neural populations, and thus can help characterize the nature of those populations, such as the number and tuning of the underlying mechanisms (Barlow and Hill, 1963; Kohn, 2007; Webster, 2015). Face aftereffects appear to at least partly reflect processes at higher and possibly face-specific levels of coding, as evidenced by the finding that the aftereffects generalize across changes in the size (Zhao and Chubb, 2001; Rhodes et al., 2004), position (Leopold et al., 2001) or orientation of the adapting and test images (Watson and Clifford, 2003). Observations that aftereffects are larger for adaptors that are more distinctive from average norms (Robbins et al., 2007), and that adaptation to norms does not induce observable aftereffects (Webster and MacLin, 1999), have also suggested that the representation is based on a norm-based code, similar to color (Hurvich and Jameson, 1957; Webster and Leonard, 2008). However, there have also been challenges to this notion (Storrs and Arnold, 2012, 2015).

Many of the studies that have explored face adaptation involve exposures to a single face or a single category of faces. That is, studies typically present observers with face images of one type (e.g., expanded faces, male faces, young faces) and aftereffects are measured by showing that neutral test faces are biased away from the adapting category (e.g., after adapting to an expanded face, an undistorted face appears contracted (Webster and MacLin, 1999); or after adapting to a male face, an androgynous face appears more female (Webster et al., 2004)). However, for some stimulus attributes, adaptation can adjust not only to the mean of the adapting stimuli but also the variance. For example, in color vision, adapting to a temporal or spatial alternation between two colors (e.g., in a flickering field or spatial grating) results in a loss in the perceived contrast or saturation of the adapting colors (Krauskopf et al., 1982; Bradley et al., 1988; Webster and Mollon, 1991). This is in spite of the fact that the pair of colors are chosen so that the mean chromaticity equals a neutral gray, and the adaptation produces no change in the appearance of this neutral, zero-contrast stimulus. Such contrast adaptation effects are also well-known and have been widely studied in spatial vision, for example as changes in the apparent contrast of sinewave gratings (Blakemore and Nachmias, 1971; Georgeson, 1985).

Whether analogous forms of contrast adaptation occur for other stimulus dimensions, and for faces, in particular, remains unclear. Previous studies testing for them have produced only weak and indirect evidence (Spetch et al., 2004). To test for the existence of contrast adaptation in faces, we took advantage of an objective measure of face adaptation and neural response changes as measured by electroencephalography (EEG) and the emerging technique of Fast Periodic Visual Stimulation (FPVS). FPVS, also known as Steady-State Visual Evoked Potentials (SSVEP; Regan, 1966; Rossion, 2014a,b; Norcia et al., 2015), refers to a method of presenting stimuli at a fixed rate and analyzing elicited responses at that rate in the frequency domain. This technique is well-suited for adaptation studies because it allows for quantification of the neurophysiological responses associated with adaptation in a coherent frequency-domain response rather than across multiple ERP components (e.g., Retter and Rossion, 2016b). Additionally, FPVS promises to be a reliable technique due to its objectivity, with responses analyzed at an experimenter-defined frequency; its implicit nature, not requiring any stimulus-related tasks; its resistance to artifacts, allowing for the production of high signal-to-noise ratios with relatively few trials; and the relative ease with which the data is analyzed and interpreted (Rossion, 2014a,b).

Retter and Rossion (2016b) previously used FPVS to measure the effect of adaptation on neural responses to facial identity. Within their paradigm (derived from Tyler and Kaitz, 1977; Ales and Norcia, 2009) a face and its anti-face (i.e., an original face’s configural opposite, see Leopold et al., 2001) are sequentially presented at a rate of 6 Hz and responses are observed only at the presentation rate, indicating both identities produced symmetric responses. When the same test sequences were preceded by adaptation to one of the identities, additional responses at the image alternation rate (3 Hz) were observed, indicating that an asymmetry in the responses to the two identities had been induced. Substantial size changes in the stimuli at each presentation, as well as the location of maximal responses at 3 Hz over right occipito-temporal regions, suggest these effects may reflect adaptation at high-level and possibly face-selective visual areas. This position was further supported by a follow-up study showing that adapting and testing with inverted face images, a transformation known to disrupt face processing (Farah et al., 1995; Kanwisher et al., 1998), did not produce significant asymmetry effects (Retter and Rossion, 2017). Furthermore, adapting to the average of the test-adapt face pair also did not result in a signal at 3 Hz. Collectively, these results suggest that exposure to one of the faces altered responses at higher visual stages to the adapting face, demonstrating a neural signature of behavioral face aftereffects.

**Figure 1:**
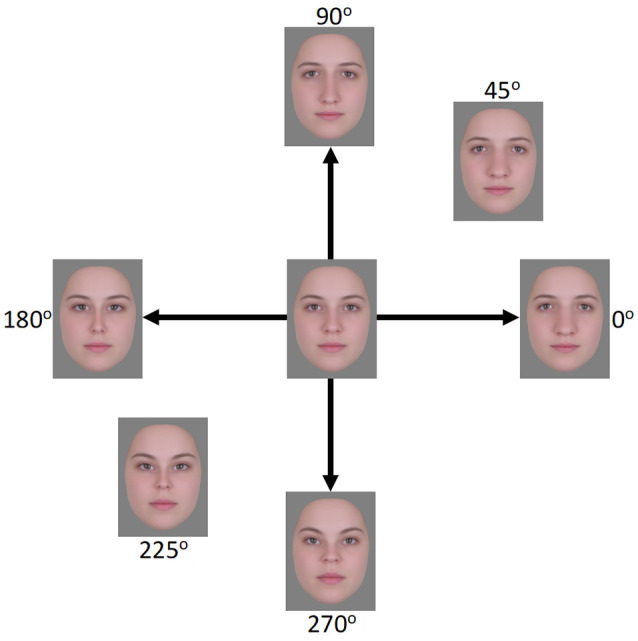
Face images used in the EEG experiment. The horizontal axis (0°–180°) represents expansion/contraction along the horizontal plane of the face while the vertical axis (90°–270°) represents expansion/contraction along the vertical plane of the face.

For the current study, we expanded on Retter and Rossion (2016b) paradigm to include four test faces rather than two. These faces formed complementary pairs of distortions, with one pair expanded or contracted along the vertical axis, and the second pair expanded or contracted along the horizontal axis (see Figure 1). We then adapted participants to one or the other pair to examine how this altered the relative responses to the two pairs. Because the two pairs share the same mean (the undistorted face), a change in the relative responses to the face pairs would reveal an adaptation adjustment that occurs independent of the mean, and instead would potentially reflect an adjustment to the variance or contrast in the face distribution.

## EEG Adaptation

### Methods

#### Participants

Fifteen (8 male) young adults (mean age = 23.73, *SD* = 3.67) from the University of Nevada, Reno, took part in the experiment. They included author OG. Observers provided signed, informed consent before participating in the experiment, which was approved by the University of Nevada, Reno’s Institutional Review Board, and conducted in accordance with the Code of Ethics of the World Medical Association (Declaration of Helsinki). Optical corrections were worn where applicable.

#### Stimuli

Stimuli were derived from 24 frontal-view images of Caucasian females with neutral expressions^1^. The images were combined using Abrosoft Fantamorph 5 (USA) to create a single “prototype” face for the set. This was achieved following standard morphing procedures in which a series of landmark points were used to denote the shape and location of features. Pixels at these points were then warped to the average location of all images. Using Adobe Photoshop CS5 (Adobe, USA), the average image was cropped following the jaw and hairline to remove all information outside of the face. The image was then re-sized to a width of 255 pixels, measured across the midline of the eyes, and shown on a gray background. Distorted versions were created by expanding or contracting the image around the midpoint of the nose using custom software created in MATLAB (Mathworks, USA) and described in Webster and MacLin (1999). Pixels were relocated along x, y coordinates at different amplitudes while being confined by a Gaussian envelope such that distortions were largest around the midpoint of the image and taper towards the edges (see Figure 1). Test images were formed by distorting the image along the four cardinal directions (0°, 90°, 180°, and 270°, corresponding to horizontal expansion, vertical expansion, horizontal contraction, and vertical contraction, respectively; see Figure 1). Adapting images were selected for three separate conditions. In the “complementary pair” condition, the adapting images formed opposite pairs along either the vertical (90°–270°) or horizontal (0°–180°) axes. This condition forms the primary focus of the study and was designed to induce contrast adaptation. The remaining two conditions were designed to induce the more traditional “mean shift” form of face adaptation. In the “orthogonal pair” condition, images came from two orthogonal axes, either 0° and 90° (both expanded) or 180° and 270° (both contracted). In the “orthogonal single” condition, a single adapting image was used, which was the mean of each of the two orthogonal image sets from the “orthogonal pair” condition. For the 0° and 90° pair, this was an image at 45°, while for the 180° and 270° pair this was an image at 225°. Images were presented on a NEC AccuSync 120 cathode ray tube (CRT) monitor with a screen size of 450 × 350 mm, a working resolution of 1,280 × 960 pixels, and a refresh rate of 60 Hz. At a viewing distance of approximately 57 cm, the images subtended a visual angle of 8.1 degrees, measured across the midline of the eyes.

#### Procedure

The experiment took place in a darkened room and the session for each participant lasted approximately 1.5 h, including 30 min of preparation and 1 h of recording. Images were presented using the FPVS technique (Rossion, 2014a,b) and custom software running over Java 8 (Oracle, USA). A single trial consisted of a 40 s sequence in which stimuli were shown at a fixed rate of six images per second (6 Hz) by means of a square wave modulation at a 100% duty cycle. To reduce the potential impact of low-level properties of the images contributing to possible observed effects, the size of the images varied randomly across five steps between 90% and 110% of the original image size at each stimulus presentation cycle (Dzhelyova and Rossion, 2014). During each trial, participants were required to fixate on a small cross in the center of the screen. To help ensure attention was maintained, the fixation cross would briefly change to a square eight times during each trial at random intervals, and participants were required to press a key to indicate they saw the change.

The experiment comprised a total of four conditions (three adaptation conditions and one non-adaptation condition), with eight trials in each. Following a similar procedure as Retter and Rossion (2016b), for the adaptation conditions, the 40 s sequence was divided into an initial 20 s adaptation phase and subsequent 20 s test phase. Test phases began immediately after adaptation phases, resulting in a continual sequence of faces during the 40 s trials. During the adaptation phase, two of the adapting images alternated with each other at the presentation rate of 6 Hz, except in the “orthogonal single” condition, in which a single adapting image was repeated. For conditions containing two adapting images, four versions of the adapting sequences were created such that each image in a pair was seen first in the sequence (e.g., 0° followed by 180°, 180° followed by 0°, 90° followed by 270°, and 270° followed by 90°). Each sequence was seen twice, resulting in eight trials per condition. For the complementary pair condition, the test phase began with one of the cardinal directions and moved through the sequence in a counter-clockwise direction (see Figure 1). For example, if the sequence began with the image at 0°, the next image would be at 90°, followed by 180°, then 270°, and then back to 0°, at which point the loop would begin again. Adapting sequences and test sequences were matched such that the first image seen in an adaptation sequence was always the first seen in the test sequence. For the test sequence described above, the adaptation sequence would comprise an alternation beginning with the image at 0°, followed by the image at 180°. For the non-adaptation condition, the test sequences were repeated without a preceding adaptation sequence. For the two orthogonal conditions, the test sequences were slightly different. For these conditions, two images from the same axis were shown in succession followed by two images from the other axis. For example, if the test sequence began with the image at 0°, the next image would be at 180°, followed by the image at 90°, then 270°, and back to 0°. Adapting and test sequences were again matched such that the first image seen in a test sequence was also the first image seen in the adapting sequence or a product of one of these images in the case of the orthogonal single condition. Counterbalancing the order of images in test sequences and yet matching them to adapting sequences allowed us to reduce any possible inherent differences in the ways the visual system processes the images, which could contribute to asymmetries in the EEG signal separate from the effects of adaptation. Inter-trial-intervals (ITIs) varied, with the beginning of new trials initiated by participants using a key press. ITIs were not tightly controlled as the effects of adaptation and the presence of the 3 Hz signal have been shown to dissipate after the first 3.33 s of the test phase and thus before the beginning of the next trial (Retter and Rossion, 2017).

#### EEG Acquisition

The data were recorded using a BioSemi ActiveTwo system with a 128 Ag-AgCl active-electrode array (BioSemi B.V., Amsterdam, Netherlands; for exact position coordinates, see BioSemi website^2^, for a conversion of these coordinates to a more standard 10-5 nomenclature (Oostenveld and Praamstra, 2001), see Rossion et al., 2015). Electrode offsets were kept below 40 mV, referenced to the common mode sense (CMS) and driven right leg (DRL) loop. Four additional electrodes were used to record vertical and horizontal electrooculogram (EOG): two electrodes were placed above and below participants’ right eye and two were placed lateral to the external canthi. The EEG and EOG were digitized at a sampling rate of 2,048 Hz and then down-sampled to 512 Hz.

#### Analysis

The recorded EEG was analyzed using Letswave 5, an open-source toolbox^3^, running over MATLAB R2013b (MathWorks, USA).

##### Preprocessing

Data files for each participant were first filtered using a fourth order zero-phase Butterworth band-pass filter, with cutoff values of 0.1–120 Hz. A Fast Fourier Transform (FFT) multi-notch filter with a width of 0.5 Hz was also applied to remove electrical noise at three harmonics of 60 Hz. The data were then segmented by trial, including 1 s before and after the beginning of stimulation. To correct for artifacts caused by eye blinks, independent component analysis (ICA) with a square matrix was applied (Hyvarinen and Oja, 2000). A single component was removed for three participants who blinked more than 0.18 times/s on average during the 20 s test sequences. This cutoff is similar to previous studies that have used similar experimental designs (e.g., Retter and Rossion, 2016b). Channels containing artifacts across multiple trials were replaced with the average of two to four neighboring channels. All channels were then re-referenced to the common average. For each subject, adaptation trials were re-segmented to exclude the initial 20 s adaptation sequence. Non-adaptation trials were re-segmented to only include the first 20 s of the sequence. Trials were then averaged within each condition.

##### Frequency Domain Analysis

An FFT was computed for each subject, condition, and channel, transforming the EEG data into separate frequency-domain amplitude and phase spectra. The amplitude data were then grand averaged across all subjects. Recordings were analyzed using a right occipito-temporal (ROT) region of interest (ROI), comprising electrodes PO8, PO10, PO12, P10, and P8 (Retter and Rossion, 2016b). The presence of a significant response at the frequency of interest was determined by *z*-scores (*z* = (x-baseline)/standard deviation of the baseline). Baselines were defined as the 20 bins surrounding the bin of interest (x), excluding the immediately adjacent bins (Srinivasan et al., 1999; Rossion et al., 2012). When displaying the amplitude spectra and comparing differences in amplitude across conditions, baseline corrections were applied to account for differences in baseline noise across participants and across the frequency spectrum within participants. This took the form of a baseline subtraction in which the average of the 20 surrounding bins, excluding the immediately adjacent bins and the local maximum and minimum amplitude bins, was subtracted from the bin of interest (x’= x-baseline). When comparing differences in amplitude, the sum of baseline-subtracted harmonics of the frequency of interest was also computed (Retter et al., 2021). For responses at 3 Hz the even harmonics were not included as these correspond with the presentation rate of 6 Hz. The number of harmonics summed was determined by the condition with the highest continuation of significant harmonics.

##### Time Domain Analysis

While the effect of adaptation in introducing asymmetries in responses can be clearly observed by analyzing response amplitudes at 3 Hz in the frequency domain, the source of these asymmetries is less clear. For example, asymmetries could arise from a reduction in the amplitude of the response to the adapted face relative to the non-adapted face, similar to adaptation in fMRI (Grill-Spector et al., 1999), or* vice versa*. To examine this, we would ideally consider the responses to each stimulus in the time domain of the EEG recording. However, responses to face stimuli presented at 6 Hz are overlapping in time, such that the responses to each stimulus are not distinct (Retter and Rossion, 2016b). Therefore, following analysis procedures used in Gwinn et al. (2018), for each condition and participant we instead modeled the effect of the 3 Hz response on the amplitude of the first and second cycle of the 6 Hz response in the time domain, with the assumption that these 6 Hz cycles may more closely reflect responses to the first (adapted) and second (non-adapted) images in a sequence, respectively. Beginning with the data averaged across trials described at the end of pre-processing, the cosine phase and amplitude of the 3 Hz and 6 Hz responses were calculated and plotted across a 334 ms time window (i.e., one cycle of the 3 Hz wave and two cycles of the 6 Hz wave). These two waveforms were then summed and the amplitude of the first cycle (0–167 ms) was compared to the amplitude of the second cycle (167–334 ms). A 50 ms delay from image onset was included when defining the beginning of the first cycle as this may be the earliest time point at which responses to faces can be observed (Seeck et al., 1997). The phase of the 3 Hz wave relative to the 6 Hz wave predicts over which time periods the 6 Hz response would be increased or decreased, with the amount of change in the 6 Hz wave determined by the amplitude of the 3 Hz response. Results from this analysis should be interpreted with a degree of caution. While differences in amplitudes across the waveforms may be accurately quantified, how these differences relate to the specific images is less clear. Unlike ERP studies or other FPVS studies in which there is a larger amount of time in between stimuli of interest (Dzhelyova et al., 2016; Retter and Rossion, 2016a), presenting facial images at a rate of 6 Hz means that we are measuring numerous overlapping responses, which makes it necessary to approximate distinct responses for single images.

### Results

#### Frequency Domain

**Figure 2:**
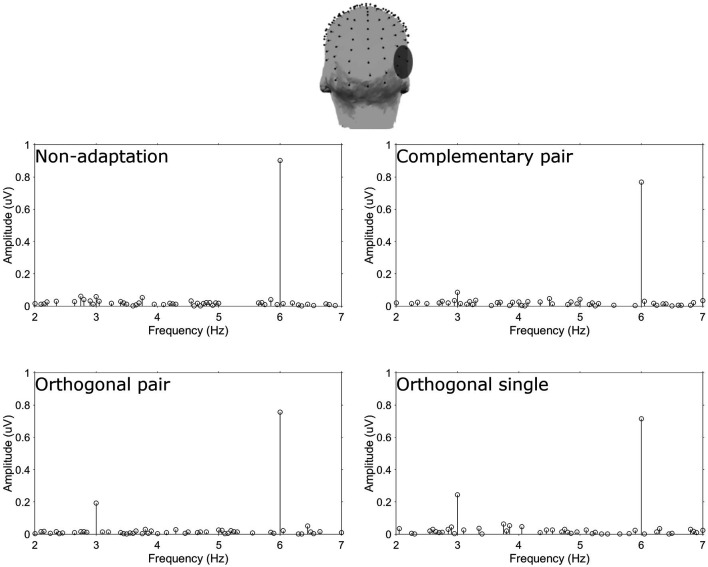
Top head map shows the electrodes comprising the ROT ROI. Plots show baseline subtracted amplitude spectra for the four conditions.

**Figure 3:**
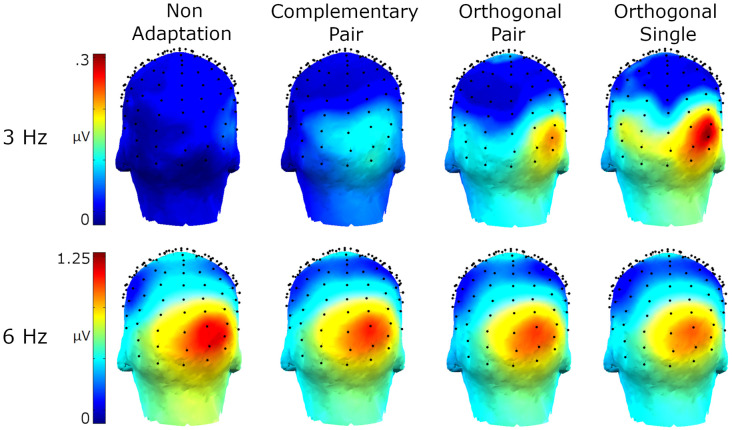
Head maps showing baseline subtracted amplitudes at 3 Hz and 6 Hz for the four conditions.

Amplitude spectra are presented in Figure 2 and topographies in Figure 3. In these figures, large responses at the image presentation rate of 6 Hz can be seen across all conditions. Conversely, responses at 3 Hz appear to only be present in the three adaptation conditions and absent in the non-adaptation condition. Inspection of the *z*-scores (see Table 1) confirmed that significant responses at 3 Hz were only present in the adaptation conditions. This indicates that without prior adaptation, responses to each image in the test sequences were symmetrical. Following adaptation, an asymmetry is introduced, resulting in the additional signals at 3 Hz.

**Table 1:** *Z*-scores for 3 Hz signals in each condition, over the ROT ROI.

Non-adaptation	Complementary pair	Orthogonal pair	Orthogonal single
1.55	2.86*	9.85**	7.01**

Notes: *p < 0.01, **p < 0.0001.

**Figure 4:**
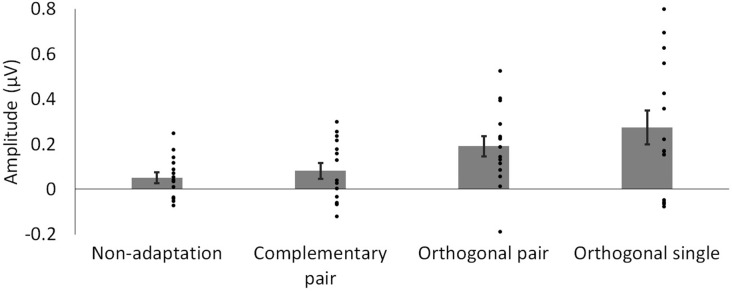
Mean baseline-subtracted amplitudes for responses at 3 Hz, summed across harmonics. Dots show amplitudes for individual participants. Error bars show SEM.

To compare the magnitude of the adaptation-induced response asymmetries across conditions, a sum of harmonics was calculated. Significant harmonics with *p* < 0.05 were observed up to the 3rd harmonic (9 Hz) in the orthogonal single condition. Baseline-subtracted amplitudes for the first three harmonics, excluding the second as this corresponds to the base stimulation frequency (6 Hz), were summed for each condition separately. The average of these summations across participants is shown for each condition in Figure 4. Two paired-samples *t*-tests were used to separately compare the magnitude of responses in the complementary pair vs. orthogonal pair conditions, and the orthogonal pair vs. orthogonal single conditions. These analyses showed that response asymmetries in the orthogonal pair condition (*M* = 0.19, *SD* = 0.17) were significantly greater than those in the complementary pair condition (*M* = 0.08, *SD* = 0.14; *t*_14_ = 2.22, *p* = 0.043, *d* = 0.70). Conversely, these same response asymmetries in the orthogonal pair condition were not significantly different to those in the orthogonal single condition (*M* = 0.27, *SD* = 0.29; *t*_14_ = 1.05, *p* = 0.314, *d* = 0.34).

#### Time Domain

**Figure 5:**
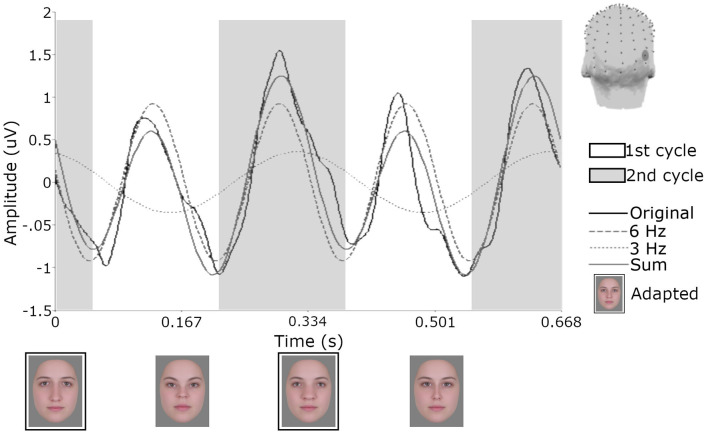
Responses from one participant for electrode PO10 following adaptation to the orthogonal single condition. Across the 20 s test sequence data were segmented in 29 epochs of 670 ms and then averaged. Shaded areas demonstrate how the signal would be defined as first and second cycles of the 6 Hz waveform and the influence of the 3 Hz signal. Face images with black borders are constituents of the single adapting image. The head model shows the location of electrode PO10.

To first visualize the recordings in the time domain, a more conservative Butterworth low-pass filter was applied to the data averaged across trials described at the end of pre-processing. This fourth order filter comprised a cutoff of 30 Hz, which is more typical of a filter used in ERP studies of face perception (e.g., Jacques et al., 2007). Data were then segmented into 670 ms epochs to encapsulate the presentation of and responses to a full four-face cycle of test images (since each image was displayed for 167 ms). The 29 epochs within each condition were then averaged separately for each participant. The resulting waveform for one participant for the orthogonal single condition can be seen in Figure 5 for electrode PO10. This electrode was chosen as it showed the largest responses at 3 Hz. Data from a single participant is shown to avoid averaging over differences in latency across participants (for examples of such variance, see Retter and Rossion, 2016b). Note that these transformations were only for the purposes of visualizing the recordings and the subsequently reported analyses were performed using the original trial-averaged data described at the end of pre-processing.

**Figure 6:**
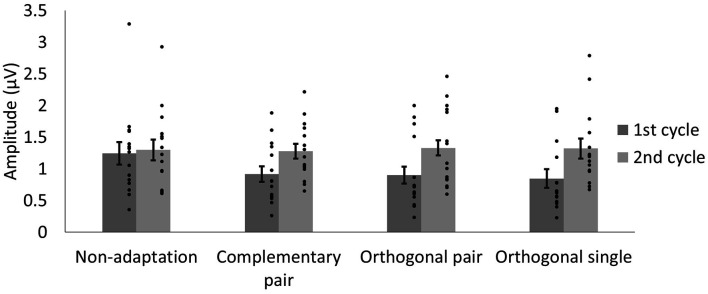
Average amplitudes of the first and second 6 Hz cycles, modeled from the sum of the 3 Hz and 6 Hz signal data for each condition. The lower amplitude of the first cycles in the adaptation conditions may reflect responses driven by faces more similar to the adaptation images. Dots show amplitudes for individual participants. Error bars indicate SEM.

To quantify differences in amplitude across the waveforms, we modeled the sum of the 3 Hz and 6 Hz responses for each subject and condition across a 334 ms time window and compared the amplitude of the 50-ms delayed first cycle of the wave (50–217 ms) to the second cycle (217–384 ms), as described in “Methods” section “Time domain analysis”. In Figure 5 the shaded areas illustrate the effective time windows for the two 6 Hz cycles across a 670 ms time window. Note that for the following analyses this was calculated for the full 20 s test sequence. Given the relative cosine phase difference between the 3 Hz and 6 Hz responses, the 3 Hz wave will be expected to have introduced an asymmetry in the 6 Hz response by enhancement of either the first or second 6 Hz cycle. Note that due to our sequence design, explained in section “Procedure”, the facial image presented within the first cycle was always more similar to one of the adaptation images compared to the face in the second cycle. The amplitude of the first and the second 6 Hz cycles averaged across participants can be seen for each condition in Figure 6. The amplitude of the two 6 Hz cycles appears similar for the non-adaptation condition in which the signal at 3 Hz was found to be non-significant, whereas clearer differences between the amplitudes of the two cycles can be seen for the remaining three adaptation conditions, in which significant responses at 3 Hz were observed. These differences were formally analyzed using four paired-samples *t*-tests. Analyses confirmed the difference between cycles was significant for all conditions except the non-adaptation condition (see Table 2), with the second cycle response amplitude always appearing larger (see Figure 6). As previously mentioned, analyzing the present data in the time domain can be problematic as the recorded signals at 3 Hz and 6 Hz likely represent overlapping responses. However, if we take responses to be associated more with the image primarily presented in the same time window than the image not being presented, these results indicate a reduction in the responses to faces more similar to the adaptation images.

**Table 2:** *t* and *d* statistics for comparison of the amplitudes of the first and second cycles of the sum of the 3 Hz and 6 Hz signal for each condition.

	Non-adaptation	Complementary pair	Orthogonal pair	Orthogonal single
*t*	0.76	4.43*	5.4**	8.51**
*d*	0.08	0.79	0.72	0.84**

Notes: *p < 0.001, **p < 0.0001.

## Behavioral Adaptation

**Figure 7:**
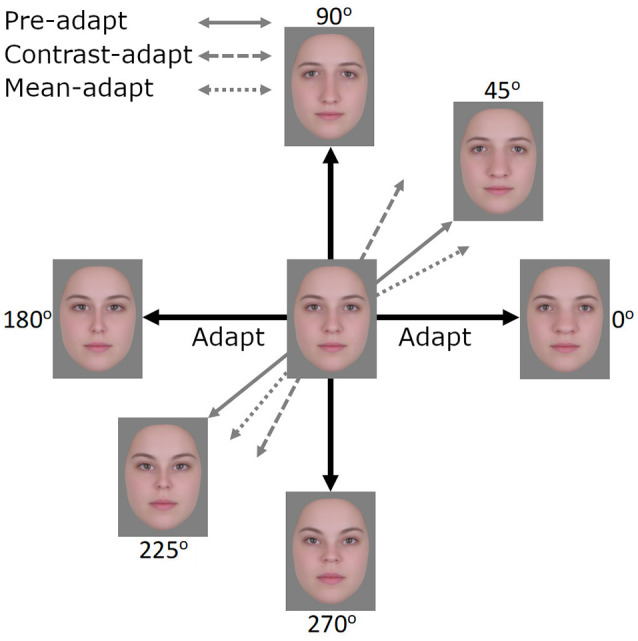
After adapting to images along the 0°–180° axis, the expected effects of contrast adaptation would be observed as images along the 45°–225° axis appearing more similar to images along the 90°–270° axis, akin to a tilt aftereffect. If mean adaptation were to occur, perhaps in the form of the neutral point shifting towards the face at 180°, then the face at 45° would appear less similar to the 90°–270° axis and the face at 225° more similar to the 90°–270° axis.

In the preceding experiments, we presented evidence for contrast adaptation in face coding, using EEG. We next examined whether the effects of this contrast adaptation can also be observed behaviorally. To assess this, we explored a different and potentially sensitive measure of face aftereffects based on the logic of tilt aftereffects (Gibson and Radner, 1937). Adapting to a tilted line produces a bias in the perceived orientation of nearby lines. We tested for an analogy to these aftereffects for faces. In this case, adapting to one axis in the space (e.g., the horizontal 0–180° axis) should selectively reduce sensitivity to the face dimension being encoded along this axis (i.e., horizontal expansion/contraction) while sensitivity along the vertical axis remains unaffected. This imbalance in sensitivity would then rotate the appearance of other faces toward the non-adapted vertical axis (Figure 7). Importantly, if this is equivalent to a contrast aftereffect, then the opposite poles of a test axis should rotate in the same way. That is, both clockwise or both counterclockwise away from the adapting axis. For example, after adapting to the 0–180° axis, faces along the 45–225° axis should show a counterclockwise bias so that the 45° face appears more like the 90° face while the 225° face appears more like the 270° face. As both the horizontal and vertical axes share the same mean and this mean represents the neutral point (i.e., neither expanded nor contracted), adaptation resulting in a mean bias should not be possible. However, if a mean bias was induced, perhaps in the form of the neutral point becoming more horizontally contracted and shifting towards the 180° face, then test faces at 45° and 225° should rotate in opposite ways (i.e., the 45° face will be biased away from the vertical axis and the 225° biased towards the vertical axis). A similar logic has also been applied to contrast adaptation and tilt aftereffects in color space (Webster and Mollon, 1991). Here we asked whether there are also tilt aftereffects in a configural face space.

### Methods

#### Participants

The 11 participants included authors OG and SO and nine (six male) students from the University of Nevada, Reno (mean age = 28.89, *SD* = 4.01). They provided signed, informed consent before participating in the experiment, which was approved by the University of Nevada, Reno’s Institutional Review Board, and conducted in accordance with the Code of Ethics of the World Medical Association (Declaration of Helsinki). Optical corrections were worn where applicable.

#### Stimuli

Stimuli were created following the same procedures described in the previous section “Stimuli”. Adapting stimuli were images along the vertical (90°–270°) and horizontal (0°–180°) axes (see Figure 1). However, in this case, we adapted observers to multiple levels along a given face axis rather than a single pair of faces, as in the EEG experiments. This was done to more closely replicate the methods employed by Webster and Mollon (1991), but can be expected to induce the same “balanced” state of adaptation that is achieved using two complementary stimuli (i.e., in both cases the opponent pools of neurons are being stimulated equally). To create these multiple levels, the maximum distortion level was doubled to produce more perceivable differences between the 20 images that spanned each axis in equal steps (40 total; the neutral face was not included). Test stimuli in this case also differed and were formed by images that fell along a circle of constant radius within the space. This was done by keeping the distortion magnitude constant while varying the angle of the distortion from 0° to 90° or 180° to 270° in 5° steps (38 images total). The distortion magnitude of the test images was half that of the adapting images (i.e., they were the same magnitude as used in the EEG experiments). Images were presented on a Cambridge Research Systems Display++ LCD monitor at a working resolution of 1,920 × 1,080 pixels and a refresh rate of 120 Hz. At a viewing distance of approximately 57 cm, the images subtended 9.2 degrees of visual angle, measured across the midline of the eyes.

#### Procedure

Experimental sessions began with an initial 2.5 min adaptation phase in which participants were simultaneously adapted to images along one axis in one visual field and the other axis in the other field (e.g., 0°–180° in the left field and 90°–270° in the right field). Images were presented approximately 8.5 degrees to the left and right of a central fixation cross, measured from the center of the image. Participants were instructed to maintain fixation on the central cross throughout adaptation and test phases. Images were shown sequentially on a continual loop in a pseudo-random order, ensuring each image was seen an equal number of times and the last three images seen at the end of the sequence were never the three maximum expansion or contraction amplitudes. This was done to reduce possible recency contrast or assimilation effects. Each image remained on the screen for 500 ms with an inter-stimulus interval (ISI) of 0 ms. Additional 10 s “top up” adaptation sequences were shown after each trial, with each of the 20 faces shown for 500 ms each. Following the initial adaptation phase and an ISI of 500 ms, participants viewed two test images presented on either side of the fixation cross in the same locations as the adapting images. In a single testing session, these images were only from the 0°–90° quadrant or the 180°–270° quadrant. For these test images, participants were required to rate which of the two (left or right) appeared more horizontally expanded by pressing the corresponding mouse button (left or right). The order of presentation of the test images was determined using a double interleaved staircase. Interleaves began with the largest possible difference between the two images. For example, one interleave would start with the 0° image on the left and the 90° image on the right, and the other interleave would start with the 0° image on the right and the 90° image on the left. If a participant rated the right image as appearing more expanded, on the next trial a more contracted version was shown on the right and a more expanded version was shown on the left. Beginning with a maximum step size of an 80° adjustment (e.g., going from 0° on the left and 90° on the right, to 80° on the left and 10° on the right), this was halved after each reversal such that after the fourth reversal the minimum 5° step size was reached. A reversal was defined as a change in responding such that images in the opposite visual field now appeared more horizontally expanded. Left and right images were adjusted in tandem, such that if one was increased by 5° (e.g., from 50° to 55°) the other was decreased by 5° (e.g., from 40° to 35°). Note that on some trials it was possible for both left and right images to be identical (i.e., both 45° or 225°). As this was a forced-choice task, participants were required to select which image they nonetheless perceived as more horizontally expanded. Each interleave continued until 10 reversals had been reached. All participants reached 10 reversals within the maximum 40 trials. Points of Subjective Equality (PSE) were calculated by taking the average of the last four reversals of each interleave. A PSE indicates the angular distortion required for both left and right test images to appear equally distorted along the horizontal axis. If adapting to distortions along the horizontal vs. vertical axes were to have no effect or produce mean biases^4^, then PSEs would be similar for both conditions. If instead adaptation effectively reduces sensitivity along the adapted axis, resulting in the post-adapted image appearing more similar to the unadapted axis (as illustrated in Figure 7), the PSE is expected to shift towards the adapted axis, as it reflects the level of distortion required to null the effect. As the left and right test images were adjusted in tandem, PSEs for the left and right fields mirror each other. As such, changes in PSE as a result of adaptation will be only reported for the left field.

The experiment was run over four sessions, each separated by a minimum of 7 days to reduce potential adaptation effects carrying over between sessions. In half of the sessions, the adapting images on the left were from the horizontal axis (0°–180°) and images on the right from the vertical axis (90°–270°). In the remaining half of the sessions, this was reversed. Likewise, in half of the sessions, the test images were from the 0°–90° quadrant, while in the other half images were from 180° to 270° quadrant. Note that rather than comparing the effect of adaptation to a baseline we will be comparing the effect of adapting to one axis compared to the other.

### Results

**Figure 8:**
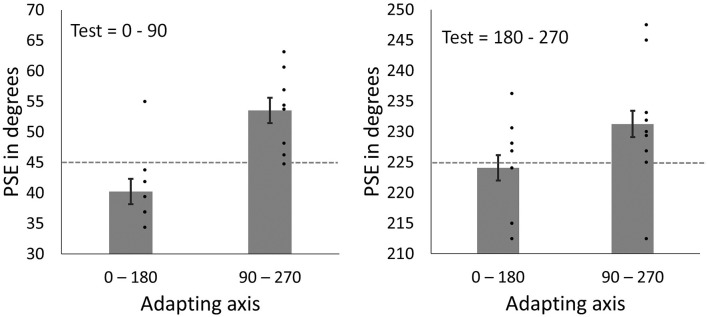
PSEs for test faces from the 0°–90° quadrant (left) and for test face from 180° to 270° quadrant (right) after adapting to horizontal (0°–180°) and vertical (90°–270°) axes. Dashed lines represent points of objective equality (i.e., the point at which the face images are horizontally distorted in equal magnitudes). Dots show amplitudes for individual participants. Error bars show SEM.

Mean PSEs following adaptation can be seen in Figure 8. Note that if there were no effect of adaptation, then the match (PSE) should occur when the face images are physically the same, and should fall along the 45° and 225° lines. Instead, the PSEs are displaced toward the adapting axes, consistent with nulling an aftereffect that biased the perception of the faces away from the adapting axes. As a result, for test images between 0°–90°, PSEs are larger in angle following adaptation to the vertical axis (*M* = 53.51, *SD* = 6.26) compared to PSEs following adaptation to the horizontal axis (*M* = 40.21, *SD =* 6.26). For test images between 180°–270°, PSEs are again larger following adaptation to the vertical axis (*M* = 233.59, *SD* = 8.77) compared to adaptation to the horizontal axis (*M* = 224.06, *SD* = 9.32). That is, in all cases, the PSE following adaptation shifted closer to the adapted axis. To allow for data from the two test quadrants to be analyzed together, data from the 180°–270° quadrant were re-scaled to range between 0°–90°. Data were analyzed using a 2 × 2 repeated measures ANOVA with the factors Adapting axis (vertical vs. horizontal) and Test quadrant (0°–90° vs. 180°–270°). Analyses showed a significant main effect of Adapting axis (*F*_1,8_ = 24.39, *p* = 0.001, $\eta_{\text{p}}^{2}$ = 0.75). The main effect of Test quadrant was found to be non-significant (*F*_1,8_ = 0.06, *p* = 0.816, $\eta_{\text{p}}^{2}$ = 0.01), as well as the Test quadrant*Adapting axis interaction (*F*_1, 8_ = 1.64, *p* = 0.236, $\eta_{\text{p}}^{2}$ = 0.17).

## Discussion

To summarize, we used face images distorted along vertical and horizontal axes to examine contrast adaptation in face perception. In the EEG experiment, test sequences comprised four images presented at a rate of 6 Hz. In the absence of prior adaptation, significant responses were observed only at the image presentation rate of 6 Hz, indicating symmetric responses to the four images. Adapting to complementary face pairs introduced an additional signal at 3 Hz, indicating asymmetric responses to images from the two axes. The mean of these faces is a neutral undistorted face and so these asymmetries reflect a form of contrast adaptation, as opposed to more traditional forms of face adaptation that result in a mean bias. Larger 3 Hz responses were observed following adaptation to face pairs from orthogonal axes, which would induce mean biases. The magnitude of these later 3 Hz responses was not significantly different to those following adaptation to a single face representing the mean of the orthogonal pairs.

For the behavioral experiment, we used a variant of classic tilt aftereffects (Gibson and Radner, 1937) to examine the perceptual effects of contrast adaptation in face perception. Adapting to distortions along one axis resulted in face images from intermediate axes appearing biased away from the adapted axis (i.e., nulling PSEs were closer to the adapted axis), consistent with tilt-aftereffects within face space. Importantly the two test poles rotated in the same way, arguing against a mean bias shifting the whole space in the same direction. That is, when adapting to the horizontal axis we did not find the appearance of images at 45° rotating away from the vertical axis and images at 225° rotating towards the vertical axis, which should occur if the adaptation shifted the mean neutral point to be closer to the 180° pole. These behavioral results thus lend further support to our EEG experiment in demonstrating a form of contrast adaptation for faces.

Ying and Xu (2017) previously demonstrated that adapting to a temporal sequence of face images produces behavioral aftereffects similar in size to adapting to the single mean of the sequence. Parallel neurophysiological results were observed here, perhaps indicating the same mechanism is being measured in the two approaches. Combined, these findings suggest that adaptation to faces depends on mechanisms that can encode the average value of a set of faces, similar to the way that chromatic adaptation can adjust to the average of a distribution of colors (Webster and Wilson, 2000). Similar effects have also been found for adaptation to a distribution of sizes (Corbett et al., 2012). The ability to extract the mean of a set of faces is well known from the literature on ensemble coding (Haberman and Whitney, 2007, 2009; de Fockert and Wolfenstein, 2009; Neumann et al., 2013). However, our results cannot distinguish between adapting directly to the mean as opposed to an adaptation effect that is the average of the individual response changes.

The present study shows that it is not simply the mean which is being adapted, but also the variance within a set of faces that is contributing to that mean. That is, the mean of the face pairs from the vertical axis is identical to the mean of face pairs from the horizontal axis. Adapting to this single mean would not induce an asymmetry in responses (Retter and Rossion, 2017) or bias perceptions of subsequently viewed faces (Webster and MacLin, 1999). Yet when the adapting sequence comprises variance along one of the axes, we find that responses to faces from that axis are disproportionally affected compared to responses to faces from the orthogonal axis. Complementary behavioral effects were also observed, with perceptions biased away from the adapting axis. These findings provide evidence that adaptation can adjust not only to changes in the average face but to changes in the variance.

Having demonstrated the existence of contrast adaptation in face processing, we also sought to gain some insight into the form of the underlying response changes. While the frequency domain analyses show that an asymmetry is present following adaptation, it does not reveal the direction of this asymmetry. That is, it could be the result of either an enhancement or reduction in amplitude, or increase or decrease in latency, in responses to faces from the adapted axis. To address this question we considered the relative phase of these responses. Across all adaptation conditions, the effect of summing the 3 Hz waveforms with the 6 Hz waveforms was such that the amplitude of the first cycle of the 6 Hz wave was decreased relative to the amplitude of the second cycle. Note that in this model, it is the difference between 6 Hz cycles that relates to the 3 Hz asymmetry amplitude from the frequency-domain analysis. The generally higher amplitude here may be accounted for by the focus on one channel (PO10) without a baseline noise subtraction. Adapting and test sequences were matched such that the first image seen in a test sequence was also the first image seen in the adapting sequence or a product of one of these images in the case of the single adapting image. If we take responses to be associated more with the image primarily presented in the same time window than the image not presented, these results indicate that the response changes from adaptation reflect a reduction in the amplitude of the responses to faces more similar to the adapting images. This would be consistent with predictions from models of face processing based on behavioral observations (Rhodes et al., 2005; Rhodes and Jeffery, 2006; Robbins et al., 2007) and neuroimaging studies (Grill-Spector et al., 1999; Winston et al., 2004) indicating lower response activity following adaptation, as well as for more typical faces (Loffler et al., 2005; Leopold et al., 2006).

Results from earlier EEG studies using ERP designs have been somewhat unclear regarding the effect of adaptation, also referred to as repetition priming, on components understood to reflect the processing of faces. In regards to the N170, different studies have shown that it is both sensitive (Jemel et al., 2005; Jacques and Rossion, 2006; Caharel et al., 2009) and insensitive (Schweinberger et al., 1995; Eimer, 2000; Cooper et al., 2007) to the types of within category face adaptation seen behaviorally, such as identity aftereffects (for reviews, see Rossion and Jacques, 2011; Schweinberger and Neumann, 2016). Different studies have also shown that adaptation can both increase (Herzmann et al., 2004; Jemel et al., 2005) and decrease (Itier and Taylor, 2002, 2004; Jacques and Rossion, 2006) the amplitude of the N170. More consistent results have been produced when considering the P200, a component reflecting the perceived typicality of a face (Stahl et al., 2008). In behavioral studies, faces more similar to adapting images appear more typical following adaptation (Robbins et al., 2007), and P200 amplitudes similarly indicate greater typicality of adapting images (Kloth et al., 2017). However, perhaps surprisingly, the P200 shows an increase in amplitude following adaptation rather than the reduction suggested by models of face adaptation. The inconsistencies across studies and ERP components highlights one of the advantages of the FPVS paradigm, in that the frequency-defined responses and their relative amplitude can be objectively identified. In the present study the relatively short interval between stimuli of interest makes it difficult to isolate responses to specific images, however when longer intervals are used this can be achieved with greater precision (Dzhelyova et al., 2016; Retter and Rossion, 2016a; Rossion et al., 2020). A short interval may be advantageous in that it increases competition between overlapping responses to subsequent stimuli (Keysers and Perrett, 2002; Retter and Rossion, 2016a), and thus enhances the 3 Hz asymmetry responses here. Should a future study combine a longer interval with the method for quantifying differences in amplitudes across cycles described here, the effect of adaptation on response amplitudes to specific images may be more precisely determined.

In the EEG experiment, we have accounted for the asymmetries in responses after adapting to a given axis (e.g., 0°–180°) by assuming a loss of sensitivity to that axis, or to the overall “contrast sensitivity” for that axis. However, an alternative is that the adaptation produced localized losses in sensitivity to the two adapting distortion levels (e.g., separate losses to the 0° adaptor and 180° adaptor). While the present experiments were not designed to test this alternative, we note that the latter account is more in line with the prediction of a multiple-channel model for the distortion levels, and is inconsistent with previous findings that adaptation to facial distortions instead reflects an opponent-like code (Rhodes et al., 2005). For example, adaptation to an undistorted face does not produce a perceived change in distorted test faces, an asymmetry which is inconsistent with local adaptation to the distortion level along the axis but is consistent with the prediction for adapting to a “zero-contrast” distortion (Webster and MacLin, 1999). Note that this may be different for other aspects of faces for which the underlying coding scheme may reflect a multiple-channel code.

In the behavioral experiment, we observed the two test poles rotating in the same way, arguing against a mean bias shifting the whole space in the same direction. However, an alternative account is that the aftereffects could be explained by multiple and simultaneous instances of local repulsion rather than contrast adaptation. That is, a test face at 45° is in closer proximity and more similar in appearance to adapting faces near the 0° pole. Conversely, a test face at 225° is more similar to faces at 180°. A greater similarity in appearance could be accompanied by a greater commonality in the neural populations encoding these faces. It would then follow that any changes in the response properties of these populations will have a greater effect on faces more similar in appearance. In the present study, it could be that test faces at 45° are being repulsed from 0° adaptors while test faces at 225° are being separately repulsed from 180° adaptors, giving the impression of an axis rotation. However, studies of face distortion aftereffects are generally concordant in finding that adaptation to the distortions involves a global renormalization of the space rather than a local repulsion (Webster and MacLin, 1999; Storrs and Arnold, 2012). While some aspects of face perception, such as eye gaze, appear to reflect a multiple channel coding system (Calder et al., 2008), figural distortions in faces appear to be encoded *via* a norm-based system (Robbins et al., 2007) and reflect global rather than local response changes. As such, it is unlikely that the aftereffects observed here can be explained by local repulsion to the two sides of the adapting axis, and may instead reflect a general sensitivity loss for the adapting axis.

It is perhaps worth noting that in the article by Calder et al. (2008) referenced above, participants were adapted to alternations of left/right eye gaze directions, similar to the complementary pair adaptation condition in the present EEG experiment. However, as eye gaze is understood to be encoded *via* a multiple channel system this necessarily negates the possibility of contrast adaptation as the neutral point is encoded by activation in a specifically designated channel, rather than being represented by equal activation in two opposing channels. That is, adapting to the poles of an eye gaze continuum would not produce a uniform reduction in sensitivity along that axis. Instead, a spike in sensitivity would be present around the neutral point (i.e., direct gaze).

The discovery of contrast adaptation in faces holds potentially important implications for our understanding of models of face processing. In addition to the more standard form of face aftereffects, in which perceptions are biased in a single uniform direction (e.g., Webster and MacLin, 1999), opposing contingent face aftereffects have also been observed. Contingent aftereffects involve adapting to two face categories (e.g., Asian vs. Caucasian) that also differ on a second dimension (e.g., Asian faces that have been contracted vs. Caucasian faces that have been expanded). Adapting to these faces results in simultaneous opposing aftereffects. That is, subsequently viewed Asian faces appear expanded and Caucasian faces contracted. These findings have led researchers to conclude that rather than the existence of a single norm against which all faces are encoded, separate norms are maintained for many categories of faces, including race (Jaquet et al., 2008; Gwinn and Brooks, 2013, 2015b), gender (Bestelmeyer et al., 2008; Jaquet and Rhodes, 2008), age (Little et al., 2008), and species (Little et al., 2008; Gwinn and Brooks, 2015a). However, these effects can be equally explained as tilt-like effects around a common norm and do not necessarily require the existence of multiple norms (Webster and MacLeod, 2011). That is, contingent aftereffects can be conceptualized as opposite ends of a given face dimension rotating towards an orthogonal dimension. The contrast aftereffects reported here demonstrate such a rotation is possible. The existence of a common norm would further explain why aftereffects are not observed following adaptation to a global norm (e.g., a face that is ambiguous in terms of race and gender), which under a multiple norm model should be distinctive from all single races and gender norms and induce observable aftereffects (Webster and MacLeod, 2011). While multiple category-specific norms may facilitate the coding of identity (Armann et al., 2011; Rhodes et al., 2011), they are not necessarily required for contingent aftereffects.

In the current experiment, we measured the perceptual effects of contrast adaptation in faces as a bias away from the adapting axis. However, in studies of spatial or color contrast, the effects of contrast adaptation have also been observed as a reduction in contrast sensitivity, resulting in a change in the thresholds for detecting contrasts (Blakemore and Campbell, 1969; Krauskopf et al., 1982). The expected effects of face contrast adaptation on the sensitivity to the adapted faces are less clear. In a review of visual adaptation and face perception, Webster and MacLeod (2011) note that contrast within face perception can be conceptualized in two different ways: as the magnitude of a face along a dimension (e.g., how expanded or contracted a face appears), or the physical contrast of the image (e.g., the maximum and minimum luminance values in the image). In terms of physical contrast, over very short adaptation periods (20–200 ms), thresholds for detecting faces can be increased for non-adapted faces and decreased for adapted faces, while over longer periods thresholds increase for both categories (Guo et al., 2009; Oruç and Barton, 2010). In regards to face contrast as dimension magnitude, some studies have shown a facilitation in discrimination around average or adapted faces (Rhodes et al., 2010; Oruç and Barton, 2011), while others have shown no effect (Rhodes et al., 2007; Ng et al., 2008). For studies showing facilitation effects, this may be due to a *sharpening* of the tuning curves of the neural populations sensitive to the adapted stimulus (Oruç and Barton, 2010, 2011) or a reduction in responses to common information shared by faces in a set (Rhodes et al., 2010). For our results, we did not measure detection or discrimination but rather changes in the amplitude of neural responses or biases in perceptual responses. By both measures, adaptation appeared to alter these responses in ways that are consistent with adaptation to the variance rather than the mean of the adapting images-and thus with adaptation to the face contrasts. These results, therefore, provide novel evidence for a distinct form of adaptation in the visual mechanisms processing faces.

## Conclusion

We have presented evidence for contrast adaptation in face perception. This was observed both as changes in neural responses measured using EEG, likely reflecting a reduction in sensitivity to the adapted face axis, as well as behavioral changes in the appearance of subsequently viewed faces. These findings show that in addition to the human visual system adjusting to the average face to which the observer is exposed, adaptation can also selectively adjust to the range or variance of a set of faces. These contrast adaptation effects reflect a distinct form of face adaptation and may underlie effects that have previously been interpreted in terms of contingent face aftereffects.

## Data Availability Statement

The raw data supporting the conclusions of this article will be made available by the authors, without undue reservation.

## Ethics Statement

The studies involving human participants were reviewed and approved by Institutional Review Board, University of Nevada, Reno. The patients/participants provided their written informed consent to participate in this study.

## Author Contributions

OG, TR, and MW contributed to conception and design of the study. OG, TR, and SO’N contributed to technical aspects of implementing the experiment and collecting data. OG and TR analyzed the data. OG wrote the first draft of the manuscript. All authors contributed to the article and approved the submitted version.

## Conflict of Interest

The authors declare that the research was conducted in the absence of any commercial or financial relationships that could be construed as a potential conflict of interest.

## Publisher’s Note

All claims expressed in this article are solely those of the authors and do not necessarily represent those of their affiliated organizations, or those of the publisher, the editors and the reviewers. Any product that may be evaluated in this article, or claim that may be made by its manufacturer, is not guaranteed or endorsed by the publisher.

## Footnotes

^1^Details of these images can be found in Russell (2009)

^2^ http://www.biosemi.com/headcap.htm

^3^ https://www.letswave.org/

^4^Note that both axes have the same mean, thus any mean biases would be in the same direction.
